# The Social Contract for Health and Wellness Data Sharing Needs a Trusted Standardized Consent

**DOI:** 10.1016/j.mcpdig.2023.07.008

**Published:** 2023-10-07

**Authors:** Stefanie Brückner, Toralf Kirsten, Peter Schwarz, Fabienne Cotte, Michael Tsesis, Stephen Gilbert

**Affiliations:** a Else Kröner Fresenius Center for Digital Health, Technische Universität Dresden, Dresden, Germany; b Institute for Medical Informatics, Statistics and Epidemiology, Leipzig University, Leipzig, Germany; c Medizinische Klinik III, Universitätsklinikum Carl Gustav Carus der Technischen Universität Dresden, Dresden, Germany; d Ada Health GmbH, Berlin, Germany

## Abstract

The rise of health and wellness applications has led to a new category of citizen-generated health data, which are collected through sensors and user inputs. As more parameters are measured over longer time periods, these data will gradually become more important for disease prediction, care, and research than classical clinic-generated health data. Policymakers now recognize the potential of both data types in initiatives such as the European Health Data Space, which aims to enable data sharing for patient care and research at scale. Although it could be argued that clinic-generated data come from public-funded health systems and should therefore be sharable, after depersonalization, for public service, this argument extends poorly to data from wearables and applications. We propose a new approach for standardized health consent, both broad and dynamic, to overcome consent fatigue and engage citizens in data sharing.

Due to the advances in health and wellness applications, sensor technologies, and wearables, measuring health-related parameters has never been easier and more popular. Two in 5 US adults use health applications and 35% use wearables, and at least half of them use them every day.^1^ Citizen-generated health data include symptom diaries, sensor-based measurement of glucose, heart rate, and sleep and these are critical data for disease prevention, prediction, prognosis, and management.^2^ It could be said that the center of gravity of health data is shifting from clinic-generated health data, the medical data acquired during interactions with care providers, to citizen-generated health data, a new class of big data.^3^ Clinic-generated health data were previously the only information source about a patient. Citizen-generated health data provide a new source of nonclinic generated, highly granular, and long-term data, ultimately overtaking traditional health data’s importance. Its relevance is 2-fold: (i) citizen-generated health will support better diagnosis, treatment, precision medicine, and care of the patient (primary use); and (ii) it will enrich data sets for common good purposes, such as medical research or public health monitoring (secondary use).^4^ The value of citizen-generated health data is already realised, for example, in the next generation of clinical trials, which leverage remote monitoring technology.^5^ Citizen science, the engagement of citizens in research through activities such as data sharing, is an emerging field in medicine.^6^ It gained global attention during the recent coronavirus disease 2019 pandemic, where the availability of health data generated by citizens, such as symptom reporting and wearable data, tremendously impacted disease understanding and management.^7^ Although the regulatory frameworks for their safe use are still uncertain, artificial intelligence technology, such as large language models, will likely play a critical role in collecting, processing, and summarizing these health data, making the enormous amount of data manageable for primary and secondary use.^8^

## Health Data Sharing: A Choice You Make or a Choice Made for You?

Some countries take the approach that the health care institutions that generate and hold the clinic-generated health data can choose to use and share it for research after deidentification without patient consent. This data sharing practice was recognized under limited circumstances in the General Data Protection Regulation (GDPR) and in the proposal for the European Health Data Space (EHDS).^9^^,^^10^ The proposed EHDS platform for clinic- and citizen-generated health data is designed to enable data exchange across European Union (EU) countries, and it formalizes the concept of nonconsent-based deidentified data sharing for secondary use at scale. It is proposed that authorization of secondary data sharing will be granted by national data access bodies on the basis of the legitimate interest of data requesters, such as clinical or pharmaceutical researchers, or on the basis of the execution of a task in public interest assigned by law. However, such approaches can be met with wide-scale public objection. For example, in September 2022, millions of National Health Service patients opted out of data sharing after the UK government planned to install a new scheme to share patient data with private companies.^11^ The social contract for health and wellness data sharing needs a transparent and trusted consent approach to engage people in sharing sensitive data for public good.^12^ One framing of such a social contract argues that health data are “public goods” because they are generated through public health care systems. This is controversial and limited because citizens are directly or indirectly involved in public health care financing. The argument is on weak ground when applied to citizen-generated health data sharing for secondary use without consent because the data are generated by and from the patient and their applications, often with limited involvement of health care institutions in its collection. Citizen-generated health data represent the most personal, multiparameter and time series data on individuals. Recent advances in machine learning show that pseudonymization is a limited safeguard of protection even before human error or malicious actions because new techniques could allow the reidentification of previously pseudonymized data.^13^ Consent and transparency are crucial to people’s choice to share this sensitive data, and a loss of trust in institutions is to be expected if these approaches are not followed, with likely long-term effects on public opinion.^14^

## Consent—A Broken Process That is Beyond Repair?

The current consent process for applications is well recognized to be broken.^15^ How can we increase the availability of both clinic- and citizen-generated health data for secondary use while keeping citizens in control over their data? We propose that this can be achieved through the fundamental redesign and revitalization of consent processes delivered through a framework of simple, trusted, and secure technological innovation. Much of the current resentment of consent processes developed after the introduction of the GDPR in 2018, with its requirements for the handling of personal data.^9^ The e-privacy regulation, intended to reduce “consent banners” by allowing general consent in browser settings, was planned to become effective together with the GDPR but never passed.^16^ This led to the development of websites and digital services overloaded with user-tyrannizing “pop-ups” and bad consent practices, such as nudging.^17^ It can be reasonably argued that the process has been purposely created to be tedious and broken to funnel user fast past “informed” toward blind “consent.” Even though it is mandatory to display a detailed privacy policy as part of the “informed consent,” up to 91% of users accept without reading.^18^

We set out a case that consent must be the basis for the sharing of citizen- and clinic-generated health data. Clearly, consent in health cannot be blind and based on the malpractices adopted in marketing. We propose a new standard health consent (SHC) process to maximize data sharing while keeping citizens in control over their data.

## A SHC Approach

**Figure 1:**
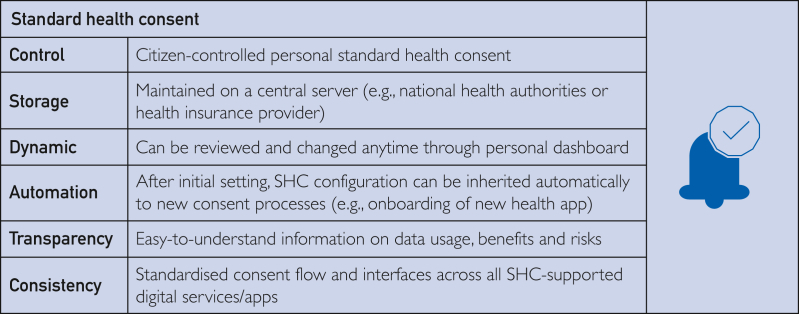
Description of standard health consent (SHC).

The proposed SHC gives citizens control of their health data while enabling primary and secondary data sharing through an easy-to-use, recognized, and standardized consent process (Figure 1). Stored on the central servers of either national health authorities or health insurance providers, the SHC would combine features of 3 overlapping recognized consent concepts and approaches: broad consent, dynamic consent, and meta-consent. Broad consent sits at the core of many research initiatives, such as biobanks, where patient consent is not bound to specific use cases, introducing flexibility for researchers to use patient samples or data for future research that is not anticipated at the time point of consent.^19^ Dynamic consent allows patients to interact continuously with consent choices and change them by using a digital platform. Meta-consent builds on broad and dynamic consent. It, too, utilizes a platform, and individuals can set upfront consent preferences (including broad and dynamic) for specific data types and research contexts.^20^ The SHC process builds on these consent concepts and provides a fully technically feasible implementation strategy with high usability for citizens and potential for universal applicability.

**Figure 2:**
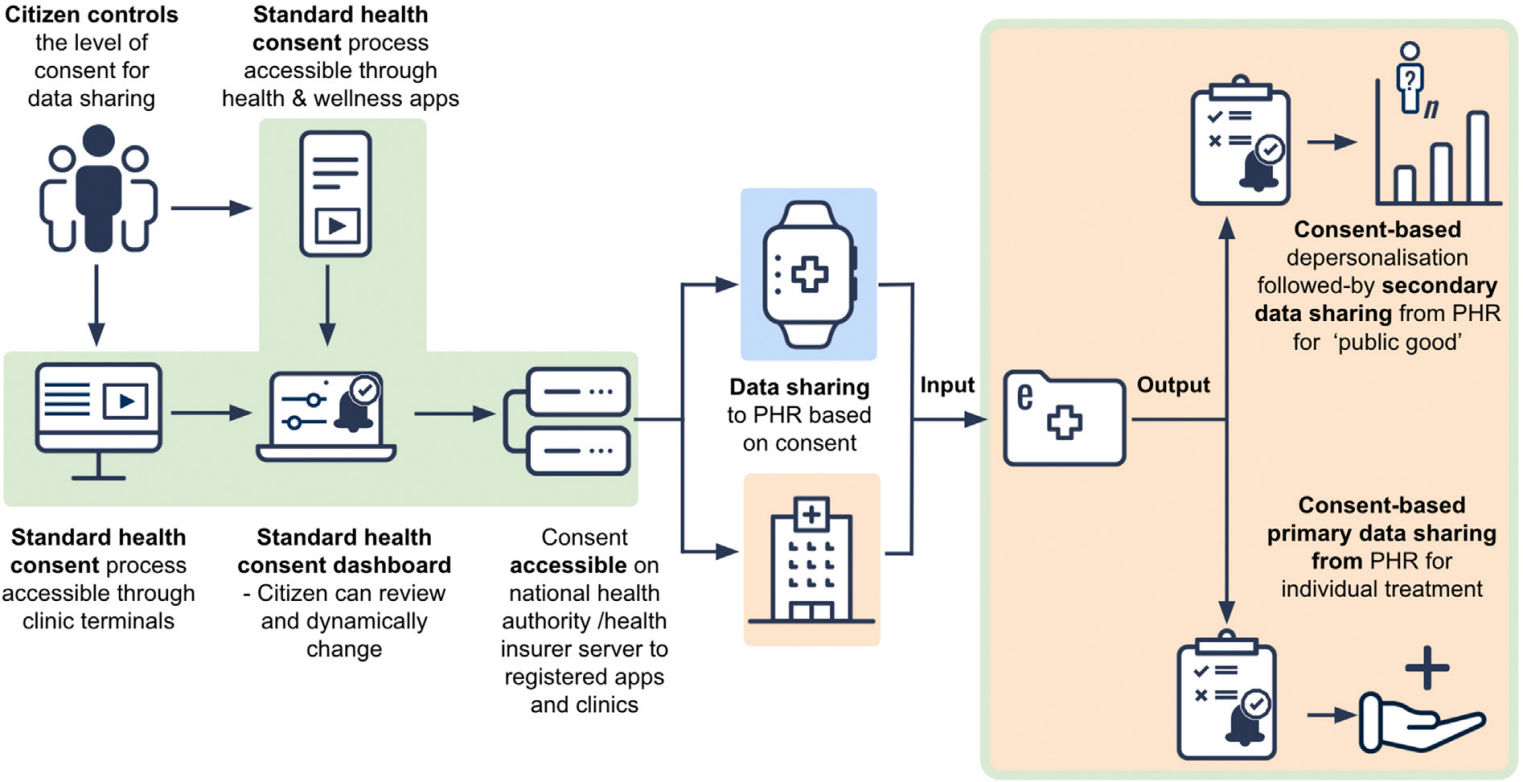
Citizen interaction with the standard health consent; the setting of consents for data inputs to the personal health record (PHR), and the setting of consents for data sharing (outputs) from the PHR for primary and secondary use. , provided via national health authority/health insurance server; , provided via application; , provided via health care provider; , provided via health care provider and national health authority/health insurance server.

In the SHC process, the citizen consent preferences would first dictate the collection of their clinic- and citizen-generated health data in their personal health record and then regulate the subsequent sharing of this data for primary and personalized secondary use. Citizens would gain access to the SHC process in 3 ways: (i) via website/application of their national health system, health insurer website, or personal health record; (ii) during a consultation with a health care provider; and (iii) through interacting with health and wellness applications that are labeled as providing the SHC (Figure 2).

**Figure 3:**
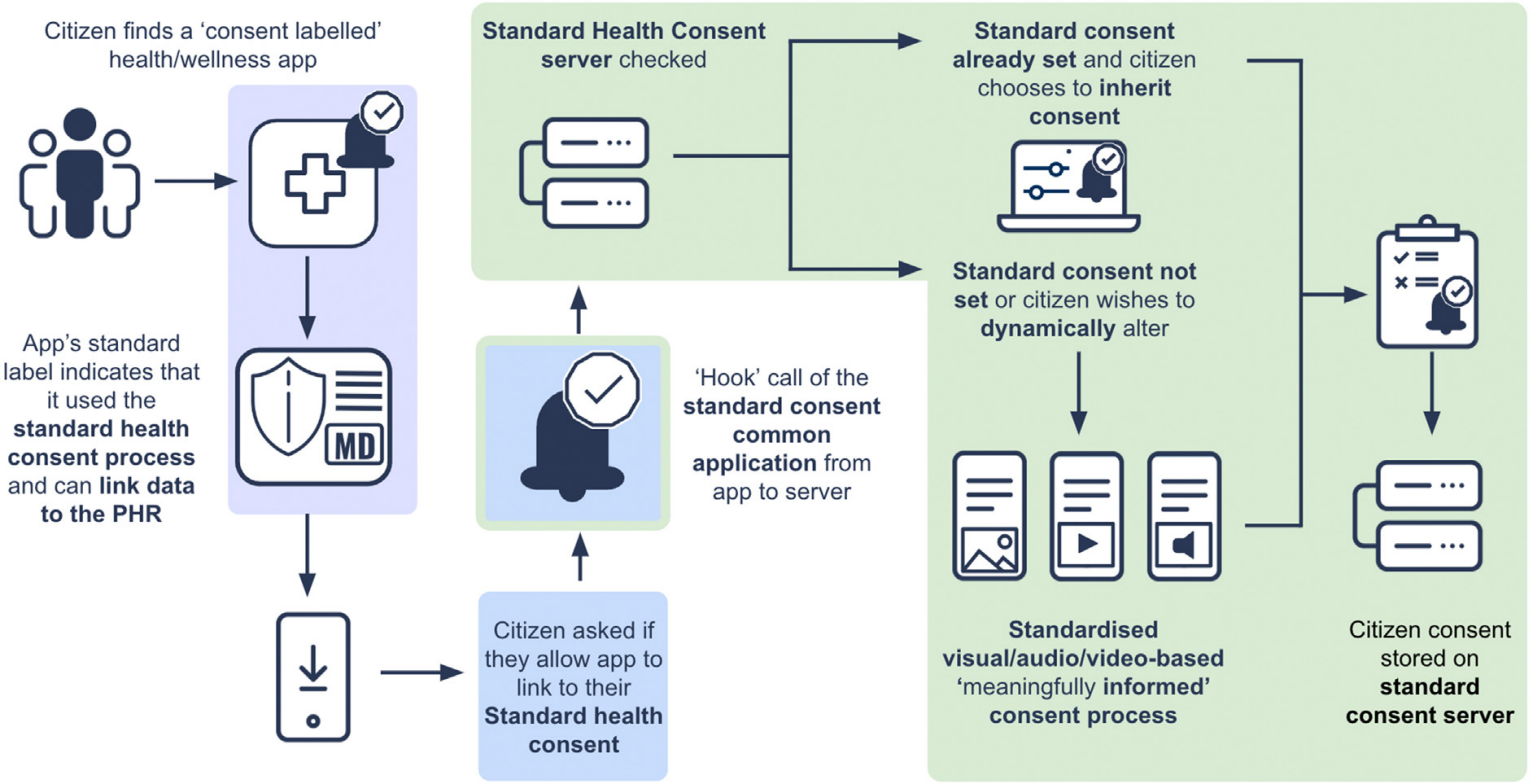
Automation, inheriting broad consent preset preferences, and on-choice dynamic editing of consents through the standard health consent during the onboarding to new health and wellness applications. , provided via application store; , provided via application; , provided via national health authority/health insurance server; , provided via application and national health authority/health insurance server. PHR, personal health record.

Independent of their entry point, citizens would always experience the standardized consent flow and interface (including all styles, labeling, and wording). First, citizens would be asked if they wish to connect to the SHC (Figure 3). If they have never previously set their consent preferences, they would be invited to set these through a simple, easy-to-understand yet comprehensive consent screen. It would inform them about the potential of data sharing for their personal health care and for wider societal benefit. They would further receive information about associated risks in a balanced manner. Standardized video, audio, or multimedia approaches would be used to explain the process, coupled with the required text descriptions of consent, meeting legal necessity but carefully optimized to ensure understandability for diverse user groups. Citizens could access and change their saved consent preferences via a highly usable dynamic dashboard on their mobile device or web browser. The standardization of the consent approach will benefit 2 main stakeholders immediately. Citizens can trust an assured, fair, and protected system, making it easy for them to participate actively. Simultaneously, physicians and researchers can trust that the data are shared on the correct legal basis that enables the safe usage of citizen-generated health data as recommended by international medical guidelines.^21^

## Is this Technically Feasible in the Short Term?

The SHC approach described is fully technically achievable using existing, secure and trusted technological identity management approaches that are commonplace in the lives of many citizens today. Citizens who use smartphones or websites for payments or authorize payments through a secure banking application have already used commercial cloud-based interfaces that manage credit card transactions (eg, Stripe and PayPal). They link out to an external application or overlay browser screen, often with highly automated 1-time password systems for authentication and security as it is used for 2-factor authorization.^22^ Similarly, users of work computers/smartphones often use cloud-based application identity providers and access management, such as Okta’s single sign-on application; most of them are based on the OAuth2, the current de facto standard for authentication and access control.^23^^,^^24^ A detailed description of the technical function is beyond the scope of this comment, but systems of this type provide the functionality for a highly secure external application (ie, the Standard Health Consent application) to interact with the developers’ health or wellness applications and for each to initiate via “hooks” the secure execution of predetermined tasks on the respective application and to allow predetermined secure sharing of structured data between the applications.

## Does GDPR Say no?

The controversy around broad consent has been growing over the past decade, with 3 main points for criticism: (i) emerging technologies for reidentification introduce previously unknown risks; (ii) the impaired process for revoking consent; and (iii) data access committees’ limitations in assessing the actual risk of individuals associated with data sharing.^19^ Despite the controversies, a broad consent approach for clinic-generated health data sharing has been recently launched by a German university–clinic initiative.^25^ The German Federal Council have called for the upcoming German Health Data Use Act to adopt both broad and dynamic consent concepts for the sharing of citizen-generated data.^26^ Legal questions remain as to whether broad and dynamic consent is the adequate legal basis for data sharing according to GDPR.^27^ We need systems that are fit for the decades ahead and the future landscape of citizen behavior. It is not okay for legislators to say, “GDPR says no.” The EHDS has a role in redefining the basis for EU data sharing, and legal refinement if and where needed should go hand in hand with technological innovation to deliver balanced benefits and freedoms to citizens.

### Summary

Some propose to replace “consent” with “no consent” as a panacea for the problems introduced through incompletely thought-out legislation and subsequent irresponsible reactions to it. Although some opponents of consent have genuinely altruistic motives toward maximizing data for research, there is also a degree of underestimation of the desire/ability of citizens to have participate consent. There are legitimate concerns that providing citizens easy-to-use tools to manage consent could lead to the large-scale refusal of consent, with the resultant biasing of data sets. To reduce the impact of refused consent, alongside trusted standardized consent processes, it will be important to engage with the public through approaches for clear and fair explanation of the social benefits that come through data sharing.^28^ This must be accompanied by clear data governance strategies for secondary use, as proposed in the EHDS.^10^ Careful quantification and stratification of consent-based biases in data could be performed, with specific public outreach approaches to proactively and fairly engage nonconsenting populations and to address their concerns. In this way, a new social contract for health data sharing based on consent and willful citizen participation could be developed. Other consent objections are financially motivated as many data-based business models are easier without the consent basis for data sharing. It is highly unlikely that a standardized consent approach, with the beneficial aspects we described, will emerge from the commercial market alone. It needs central motivation, coordination, and oversight by EU or national authorities. The proposed EHDS already suggests a common label and “approval route” for wellness applications but is entirely unclear as to what is intended as the basis for respective data sharing.^10^ The SHC approach would extend and clarify the novel and sensible EHDS approaches. Bold action is needed to replace the broken consent landscape with broad, dynamic, universal, efficient, and standardized consent approaches based on truly informed decisions. This is fully technically achievable with existing technologies, and it is legally achievable under existing and proposed EU laws or with the relatively minor adjustments as we set out. The consent approach we described is equally relevant to the international context of consent to health data sharing from health and wellness applications.

## Potential Competing Interests

Dr Gilbert declares a nonfinancial interest as an Advisory Group member of the EY-coordinated “Study on Regulatory Governance and Innovation in the field of Medical Devices” conduced on behalf of the DG SANTE of the European Commission. Dr Gilbert declares the following competing financial interests: he has or has had consulting relationships with Una Health GmbH, Lindus Health Ltd., Flo Ltd, Thymia Ltd., FORUM Institut für Management GmbH, High-Tech Gründerfonds Management GmbH, and Ada Health GmbH and holds share options in Ada Health GmbH. Dr Kirsten reports grant 16KISA106 from Bundesministerium für Bildung und Forschung. Dr Schwarz reports grants for PATH project, ADDICHRON Project, and MiHubX Project; consulting fees from International Diabetes Federation; payment or honoraria from International Diabetes Federation, Diabetes Industry, and DiGA startups; payment for expert testimony from Social court in Germany; participation on the Board of the International Diabetes Federation and the Board of the NCD Alliance; and a leadership or fiduciary role in International Diabetes Federation. Authors Brückner, Cotte, and Tsesis declare no competing financial or nonfinancial interests.
